# Effects of Dietary Chicory (*Chicorium intybus* L.) and Probiotic Blend as Natural Feed Additives on Performance Traits, Blood Biochemistry, and Gut Microbiota of Broiler Chickens

**DOI:** 10.3390/antibiotics9010005

**Published:** 2019-12-20

**Authors:** Mohammadreza Khoobani, Seyyed-Hamed Hasheminezhad, Faramin Javandel, Mehran Nosrati, Alireza Seidavi, Isam T. Kadim, Vito Laudadio, Vincenzo Tufarelli

**Affiliations:** 1 Department of Animal Science, Rasht Branch, Islamic Azad University, Rasht 41335-3516, Iran; akhoubani1352@yahoo.com (M.K.); shhashemi2016@yahoo.com (S.-H.H.); fdjavandel@yahoo.com (F.J.); nosrati@iaurasht.ac.ir (M.N.); 2 Department of Biological Sciences and Chemistry, College of Arts and Sciences, University of Nizwa, Birkat Al-Mouz, Nizwa 616, Oman; isam@unizwa.edu.om; 3 Department of DETO, Section of Veterinary Science and Animal Production, University of Bari “Aldo Moro”, Valenzano, 70010 Bari, Italy; vito.laudadio@uniba.it

**Keywords:** feed additive, growth promoter, medicinal plant, broiler

## Abstract

The experiment was designed to determine the effect of different levels of chicory (*Chicorium intybus* L.) powder and a probiotic blend (PrimaLac^®^) on productive performance, blood biochemical parameters, and ileal microbiota in broiler chickens. A total of 225 one-day-old broilers (Ross 308) were used in a completely randomized design with five experimental diets as follows: 1—basal-diet without supplements (control-group); 2—basal-diet including probiotic blend; 3— basal-diet including 0.10% chicory; 4—basal-diet including 0.15% chicory; 5—basal-diet including 0.20% chicory. At 42 days of age, representative birds per replicate were randomly selected for blood samples and carcass measurements. Results showed that the body weight gain of broilers fed the probiotic blend or 0.10% chicory was significantly (*P* < 0.05) higher than those fed on the other treatments. The abdominal fat pad was significantly (*P* < 0.05) lower in birds fed diets including chicory compared with control or probiotic. Blood triglycerides and LDL levels were reduced (*P* < 0.05) and HDL increased (*P* < 0.05) when fed probiotic or chicory whereas no significant effect on the other serum parameters was found. Broiler ileal microflora from the control group had significantly (*P* < 0.05) higher count of *E. coli* and lower *Lactobacillus* than those from the other groups. From findings, it is possible to conclude that dietary chicory powder supported positively growth performance and improved gut microbiota in broiler chickens. However, more research is needed on this subject to better understand the mode of action of feed additives used.

## 1. Introduction

Antibiotics are usually used in poultry for therapeutic, preventive, and nutritional purposes [1,2,3]. Using antibiotics for the growth promotion or prevention and treatment of infectious diseases can result in resistance among both resident bacterial pathogens and commensal organisms [4,5]. Therefore, there is increasing interest in using medicinal plants or probiotics and as natural alternatives to antibiotics for poultry production because they have the potential of inhibition against organisms improving animal performance [6,7,8,9,10,11]. 

Probiotics as live microorganisms can also improve the intestinal balance of the host livestock [12]. The mechanism of action of probiotics is mainly related to the competition for attachment sites of the intestinal mucosa, to prevent the pathogenic bacteria attachment by forming a physical obstacle [13], and also to stimulate epithelial and immune cells activities; therefore, supplementing diet with natural additives can improve poultry production. 

Chicory (*Cicorium intybus* L.) is an important medicinally perennial herb plant of the *Asteraceae* family [14], containing valuable levels of fructooligosaccharides, inulin, coumarins, flavonoids, and many vitamins [15,16]. Chicory was used as an anti-hepatotoxic, antiulcerogenic, anti-inflammatory, digestive, diuretic, depurative, alexiteric, and tonic agent [17]. In particular, inulin is one of the beneficial components in chicory that can regulate appetite and lipid-to-glucose metabolism [18]. It has also been shown that chicory can promote the growth of useful microbes [19] and inhibit gut pathogenic bacteria growth [20]. Thus, chicory could be supplemented to poultry diets to manipulate the gut microflora composition and to enhance its integrity [20], improving also broiler performance and health status by modulating lipid metabolism with hypolipidemic effects [21,22,23]. 

Therefore, the aim of this study was to evaluate the effects of chicory powder and a commercial probiotic blend as natural feed additives on the productive performance, blood biochemical parameters, and ileal microflora of broiler chickens.

## 2. Results and Discussion

### 2.1. Growth Performance 

Feed intake, body weight gain, and feed conversion ratio of broilers during the experimental period are summarized in Table 1, Table 2, and Table 3, respectively. No significant (*P* > 0.05) differences due to dietary treatment effects were observed on feed intake in the first and second growing periods, however, birds receiving diet supplemented with 0.10% chicory powder during the whole period of the experiment (1–42 days) had a higher (*P* < 0.05) feed intake than those receiving the basal or basal-diet supplemented with probiotics or 0.15–0.20% chicory. Similar results were reported by Faramarzzadeh et al. [24], who supplemented the broiler diet with 4.5% chicory powder. Live body weight gain of chickens was significantly affected by supplementing 0.10% chicory powder or probiotic blend during all rearing periods (*P* < 0.05).

Overall, the current study revealed that birds receiving diet supplemented with a probiotic blend or different levels of chicory powder during the experimental period improved significantly (*P* < 0.05) their feed conversion ratio than the other treatments. In line with the current findings, it was found that chickens fed diet supplemented with chicory powder had significantly lower feed efficiency compared to the unsupplemented group [24]. Similarly, Cabuk et al. [25] observed significant improvement in feed conversion ratio in broilers fed diet supplemented with herbal plant mixture. Further, Liu et al. [26] also reported that adding a basal diet with chicory powder significantly improved the feed conversion ratio for the first 13 days of the feeding period, which agrees with the present study. Improvement in broiler growth performance fed diet supplemented with chicory powder can be attributed to the enhancements of length, number, and surface area of intestinal villi that are paralleled with an increased digestive and absorptive capacity of jejunum [27,28]. Moreover, broiler chickens at a younger age will get more benefits than older ages by adding chicory powder to diets due to villi growth stimulation [26].

### 2.2. Carcass Traits

The effects of treatments on the relative weight of broiler carcass and non-carcass components are reported in Table 4. Apart from abdominal fat, there were no significant differences in carcass traits among dietary treatments. In particular, the abdominal fat percentage was significantly (*P* < 0.05) lower in broilers fed diet supplemented with three different levels of chicory powder than control and probiotic blend groups.

Accordingly, Pournazari et al. [10] found no effect of probiotics on carcass characteristics of broiler chickens. Furthermore, Aminzade et al. [29], Norbakht [30], Ocak et al. [31], and Mansoub [32] reported similar findings. In contrast, Panda et al. [33] and Faramarzzadeh et al. [24] found significant improvement in dressing percentage by supplementing chicory powder (3.0%) to broiler diet. The current results are also in agreement with Yusrizal and Chen [21] who found that broiler chickens fed diets supplemented with chicory fructans (1%) were heavier compared to controls. The abdominal fat accumulation in broiler chicken may be due to fat synthesis (lipogenesis) and fat catabolism via β-oxidation (lipolysis) [34]. Moreover, a positive relationship was found between adding chicory supplementation and carcass characteristics in poultry species [35,36,37]. The effect of adding chicory powder or probiotic blend to broiler diet may be due to an overall improvement of the intestinal microenvironment and to a reduction of endogenous nitrogen loss, leading to lower abdominal fat deposition in broiler chickens [21,33].

### 2.3. Blood Parameters

Table 5 summarized the effect of experimental diets on biochemical serum parameters of broilers at 42 days of age. Apart from serum triglycerides, LDL and HDL, dietary treatments did not induce any significant effect. Blood serum of broiler chicken fed control diet had significantly (*P* < 0.05) higher triglycerides and HDL concentrations than those fed chicory powder, whereas LDL level was lower by feeding experimental supplemented diets. The current findings are in agreement with those of Faramarzzadeh et al. [24], Yusrizal and Chen [21], Safamehr et al. [34] and Agazadehh et al. [38] who found that adding chicory-based fructans to broiler diet decreased also the serum total cholesterol, in addition to triglycerides and LDL in broilers. 

### 2.4. Ileal Microflora 

Data showed that adding probiotic blend and chicory powder to broiler diet had an antibacterial effect against *E. coli* while increasing *Lactobacillus* bacteria count (Table 6). The three supplementation levels of chicory powder and probiotic blend showed pronounced inhibition against *E. coli*.

In a previous study, Farrukh et al. [39] stated that chicory powder was effective against pathogenic bacteria, which in agreement with the present study. Furthermore, the antibacterial activity of chicory, and other herbal plants, was reported to counteract the growth of harmful bacteria [17,29]. The current study confirmed that chicory can be used as a natural antibacterial feed supplement, which may be due to the presence of inulin, bitter sesquiterpene lactones, coumarins, and other compounds. It has been also reported that herbs and plant extracts stimulate the growth of beneficial bacteria and minimize pathogenic bacteria activity in the poultry gut [40,41]. 

The antibacterial activity of chicory mainly depended on strains and it is dose-dependent. The present study also showed that the feed additives used contributed to reducing the growth of pathogenic bacteria such as *E. coli*, and increased the beneficial bacteria, such as lactobacilli, and similar conclusions were reported by Jain et al. [42], Ocak et al. [31], and Nobakht et al. [30]. In this respect, Lee et al. [43] reported that the presence of pathogenic bacteria population in the gut may cause the breakdown of amino acids, and thereby reduce their availability for absorption. Herbs, such as chicory, might have the ability to stimulate the production of secretions in the small intestinal mucosa, pancreas, and liver to enhance nutrients digestion and increase their availability at the intestinal brush border [34,44]. It has been also demonstrated that the main beneficial effect of probiotic blend or chicory is to induce changes in the intestinal microbiota by selective stimulation of health-promoting bacteria [45,46,47].

## 3. Materials and Methods 

### 3.1. Birds and Management 

All procedures used in this study were approved by the Animal Ethics Committee of the Islamic Azad University, Rasht Branch, Iran, in agreement with the Directive 2010/63/EU. A total of 225 one-day-old broiler chicks (Ross 308) with similar initial body weight were randomly divided into 15 experiment units of 15 birds, each with three replicates per treatment for a total of five experimental diets. The experiment was conducted at a commercial poultry farm (Khomam city, Guilan, Iran). The broilers were kept in floor pens (1.0 × 1.5 × 0.5 m) and treatment groups were equally distributed among pens. Temperature and relative humidity were maintained within the optimum range. The lighting program was 23 h light and 1 h darkness. Each pen was equipped with an individual feeder and a nipple drinker. Broilers in a pen were not able to consume feed assigned to the adjoining pen. All experiments were carried out from day 1 to day 42 of age. Birds were vaccinated against infectious bronchitis (1 and 18 days of age), Newcastle disease (1 and 18 days of age), avian influenza (1 day of age), and Gumboro disease (14 and 24 days of age). 

### 3.2. Feed Formulation

A two-phase feeding program was used; a starter diet was used from 1–21 days and a finisher diet from 22–42 days. The ingredients and nutrient composition of diets are shown in Table 7. Feed and water were supplied ad libitum throughout the experimental period. Diets were formulated to meet or exceed the National Research Council (NRC) nutrient requirements for broiler chickens. Two types of natural feed supplements were used: a multi-strain probiotic (PrimaLac^®^, Star-Labs/Forage Research Inc. Clarksdale, USA) as a lyophilized mixture containing 1 × 10^8^ CFU/g of *Lactobacillus casei*, *Lactobacillus acidophilus*, *Bifidobacterium thermophilum*, and *Enterococcus faecium* (the microbial blends and concentrations are proprietary); chicory powder purchased from a local producer (Rasht, Iran). The following dietary treatments were formulated: Treatment 1—basal-diet with no supplementation as control; Treatment 2—basal-diet supplemented with probiotic blend [following manufacturer’s instructions level: 0.900 g/kg (0.09%) from 1–14 days, 0.454 g/kg (0.0454%) from 15–28 days, 0.225 g/kg (0.0225%) from 29–42 days]; Treatment 3—basal-diet supplemented with chicory powder at 0.10%; Treatment 4—basal-diet supplemented with chicory powder at 0.15%; Treatment 5—basal-diet supplemented with chicory powder 0.20%.

### 3.3. Growth and Carcass Measurements

Mortality rate and growth performance, as feed intake, feed conversion ratio, and body weight gain, were recorded per pen at 21 and 42 days of age. After 12 h of fasting, five birds per treatment were randomly selected for carcass characteristics measurements at 42 days of age. Birds were sacrificed, plucked, and eviscerated, then the weight of the whole carcass, carcass components (drumsticks and breast), liver, gizzard, and abdominal fat were excised and individually weighed. Carcass traits components were calculated as a percentage of the preslaughter live body weight.

### 3.4. Serum Biochemical Analysis

At the end of the trial (42 days), ten broilers per group (30 birds/treatment) were selected for blood sampling. Blood samples were collected from the wing veins and centrifuged at 3000 *g* × 10 min to obtain serum. Samples were stored at −20 °C until analyzed for total protein, albumin, glucose, total cholesterol, triglycerides, high-density lipoprotein (HDL), low-density lipoprotein (LDL), and uric acid, using commercial laboratory kits (TeifAzmoon Pars Co, Tehran, Iran).

### 3.5. Ileal Microflora

For the ileal microbial count, at 42 days of age, 30 birds (two birds from each replicate) were randomly selected and killed by cervical dislocation. One gram of ileum content was sampled and transferred into sterile tubes containing 9 ml of sterile PBS and homogenized and serially diluted. A small sample (0.1 ml) from each dilution was inoculated on MRS agar (Man Rogosa Sharpe agar, Merck, 1.10660.500) was used to culture *Lactobacilli*, and Eosin Metilan-Blue (EMB, Merck, 1.01347.0500) was used for *E. coli,* then incubated under anaerobic conditions at 37 °C for 72 h. Bacterial units were counted by a colony counter and adjusted to 1 g sample. The bacteria counts were reported as log10 CFU/g of ileal digesta.

### 3.6. Statistical Analysis

The experiment was carried out in a complete randomized design with dietary feed additives as the main effects. The model assumptions of normality and homogeneity of variance were tested using Shapiro–Wilk’s and Levene’s tests, respectively. Data were subjected to analysis of variance (ANOVA) according to the General Linear Model (GLM) procedure of SAS/STAT (SAS software version 8, Institute Inc., Cary, NC, USA). The statistical model used was: Y_ijk_ = μ + T_i_ + R_ij_ + ɛ_ijk_, where: Y_ijk_ = response variables from each individual replication or pen; µ = the overall mean; T_i_ = the effect of dietary additive; R_ij_ = the inter-experimental unit (replications) error term; ɛ_ijk_ = the intra-experimental unit error term. Means were compared for significant differences using the LSMEANS option of SAS/STAT (SAS software version 8, Institute Inc., Cary, NC, USA). Statistical significance was established at *P* < 0.05.

## 4. Conclusions

Chicory powder and probiotic blend as natural feed supplements in broiler diet supported positively the growth and feed efficiency of birds, without affecting most of the blood biochemical parameters. Moreover, it is important to underline that probiotics and chicory supplementation showed a significant effect in inhibiting the growth of potentially pathogenic *E. coli*, and in enhancing the growth of beneficial *Lactobacillus* gut bacteria. Thus, this study demonstrated that natural feed additives can be successfully used as an alternative to antibiotics as growth promoters for broiler chickens.

## Figures and Tables

**Table 1:** Feed intake (g) in broilers fed experimental diets.

Item	1-21 DOA	22-42 DOA	1-42 DOA
Control	1019.5	3272.2	4293.7 ^b^
Probiotic	1025.7	3228.4	4254.1 ^b^
Chicory (0.10%)	1036.3	3279.3	4392.3 ^a^
Chicory (0.15%)	1041.0	3289.4	4330.4 ^ab^
Chicory (0.20%)	1049.1	3290.5	4255.6 ^b^
*P*-value	0.290	0.366	0.009
SEM	9.89	23.21	23.36

Means within each column with different superscript differ significantly at *P* < 0.05; DOA: days of age.

**Table 2:** Bodyweight gain (g) in broilers fed experimental diets.

Item	1-21 DOA	22-42 DOA	1-42 DOA
Control	688.7 ^c^	1331.5 ^d^	2020.2 ^c^
Probiotic	770.3 ^a^	1555.6 ^a^	2326.0 ^a^
Chicory (0.10%)	768.2 ^a^	1549.3 ^a^	2317.5 ^a^
Chicory (0.15%)	728.0 ^b^	1492.0 ^b^	2220.0 ^b^
Chicory (0.20%)	750.0 ^ab^	1439.0 ^c^	2189.1 ^b^
*P*-value	0.0002	0.0001	0.0001
SEM	8.46	13.74	17.66

Means within each column with different superscript differ significantly at *P* < 0.05; DOA: days of age.

**Table 3:** Feed conversion ratio (g/g) in broilers fed experimental diets.

Item	1-21 DOA	22-42 DOA	1-42 DOA
Control	1.48 ^a^	2.46 ^a^	2.12 ^a^
Probiotic	1.33 ^c^	2.07 ^c^	1.82 ^c^
Chicory (0.10%)	1.33 ^c^	2.16 ^b^	1.89 ^c^
Chicory (0.15%)	1.43 ^ab^	2.20 ^b^	1.95 ^b^
Chicory (0.20%)	1.40 ^bc^	2.22 ^b^	1.94 ^b^
*P*-value	0.005	0.0001	0.0001
SEM	0.022	0.025	0.018

Means within each column with different superscript differ significantly at *P* < 0.05; DOA: days of age.

**Table 4:** Carcass characteristics (%) in broilers fed different experimental diets.

Item	Eviscerated Carcass	Breast	Drumsticks	Liver	Gizzard	Abdominal Fat
Control	70.65	32.11	26.59	2.68	1.55	0.96^a^
Probiotic	70.83	31.94	26.71	2.55	1.62	0.84 ^a^
Chicory (0.10%)	70.86	31.98	26.90	2.42	1.60	0.74 ^b^
Chicory (0.15%)	70.59	32.19	26.81	2.54	1.59	0.63 ^b^
Chicory (0.20%)	70.90	32.10	26.62	2.59	1.66	0.72 ^b^
*P*-value	0.487	0.425	0.250	0.434	0.892	0.008
SEM	0.14	0.09	0.10	0.09	0.07	0.11

Means within each column with different superscript differ significantly at *P* < 0.05.

**Table 5:** Blood biochemical constitutes (mg/dl) in broilers fed experimental diets.

Item.	Total Protein	Albumin	Glucose	Total Cholesterol	Triglycerides	HDL	LDL	Uric Acid
Control	3.78	2.18	191.32	159.53	87.08 ^a^	79.33 ^a^	68.17 ^a^	5.43
Probiotic	3.83	2.30	209.89	161.11	67.49 ^b^	84.01 ^ab^	63.61 ^a^	5.64
Chicory (0.10%)	3.68	2.08	199.19	134.92	55.14 ^b^	91.66 ^b^	32.23 ^b^	5.46
Chicory (0.15%)	3.59	2.10	210.40	158.73	60.08 ^b^	93.01 ^b^	45.81 ^b^	5.69
Chicory (0.20%)	3.91	2.18	178.80	154.76	74.07 ^ab^	88.33 ^b^	51.62 ^b^	5.72
*P*-value	0.381	0.503	0.224	0.688	0.021	0.032	0.025	0.311
SEM	0.11	0.09	10.22	13.84	8.87	5.33	12.17	0.10

Means within each column with different superscript differ significantly at *P* < 0.05.

**Table 6:** Ileal microflora (log CFU/g) in broilers fed experimental diets.

Item	*E. coli*	*Lactobacillus*
Control	6.85 ^a^	6.79 ^b^
Probiotic	5.64 ^b^	7.65 ^a^
Chicory (0.10%)	5.73 ^b^	7.52 ^a^
Chicory (0.15%)	5.80 ^b^	7.70 ^a^
Chicory (0.20%)	5.99 ^b^	7.68 ^a^
*P*-value	0.0002	0.0001
SEM	0.12	0.07

Means within each column with different superscripts differ significantly at *P* < 0.05.

**Table 7:** Ingredients and chemical composition of the basal diet.

Item	Starter (1–21 Days)	Finisher (22–42 Days)
***Ingredient (%)***		
Corn	56.90	58.70
Soybean meal (44% CP)	33.10	30.00
Fish meal	3.40	3.50
Soybean oil	2.00	3.50
Dicalcium phosphate	1.55	1.10
Oyster shell	1.03	1.18
DL-methionine 98%	0.01	0.01
Vitamin mixture ^1^	0.50	0.50
Mineral mixture ^2^	0.50	0.50
NaCl	0.26	0.26
Sand	0.75	0.75
***Calculated chemical composition***		
Metabolizable energy (kcal/kg)	2910	3030
Crude protein (%)	20.1	19.0
Fat (%)	4.60	6.14
Calcium (%)	0.95	0.90
Total phosphorus (%)	1.23	1.06
Available phosphorus (%)	0.45	0.36
Methionine (%)	0.50	0.38
Lysine (%)	1.01	1.00
Methionine + Cystine (%)	0.83	0.71

^1^ Supplied per kg of mixture: 3,600,000 IU vitamin A; 800,000 IU vitamin D3; 7200 IU vitamin E; 710 mg vitamin B1; 2640 mg vitamin B2; 1176 mg vitamin B6; 400 mg vitamin B9; 6 mg vitamin B12; 800 mg vitamin K3; 3920 mg pantothenate acid; 12,000 mg niacin; 40 mg biotin; 200,000 mg choline chloride. ^2^ Supplied per kg of mixture: 40,000 mg manganese; 20,000 mg iron; 33,900 mg zinc; 4000 mg copper; 400 mg iodine; 80 mg selenium.
